# Type 2 Diabetes, Circulating Metabolites, and Calcific Aortic Valve Stenosis: A Mendelian Randomization Study

**DOI:** 10.3390/metabo14070385

**Published:** 2024-07-13

**Authors:** Rui Shen, Chengliang Pan, Guiwen Yi, Zhiyang Li, Chen Dong, Jian Yu, Jiangmei Zhang, Qian Dong, Kunwu Yu, Qiutang Zeng

**Affiliations:** 1 Department of Cardiology, Union Hospital, Tongji Medical College, Huazhong University of Science and Technology, Wuhan 430022, China; shenrui202102@163.com (R.S.); d202281928@hust.edu.cn (C.P.); yiguiwen@126.com (G.Y.); lizhiyangdoctor@163.com (Z.L.); docdongchen@163.com (C.D.); yu_j12345@163.com (J.Y.); zjm19981001@163.com (J.Z.); dongqian860322@163.com (Q.D.); 2 Hubei Key Laboratory of Biological Targeted Therapy, Union Hospital, Tongji Medical College, Huazhong University of Science and Technology, Wuhan 430022, China; 3 Hubei Provincial Engineering Research Center of Immunological Diagnosis and Therapy for Cardiovascular Diseases, Union Hospital, Tongji Medical College, Huazhong University of Science and Technology, Wuhan 430022, China

**Keywords:** type 2 diabetes, calcific aortic valve stenosis, mediation, Mendelian randomization, metabolites, blood pressure

## Abstract

Epidemiological studies have shown an association between type 2 diabetes (T2D) and calcific aortic valve stenosis (CAVS), but the potential causal relationship and underlying mechanisms remain unclear. Therefore, we conducted a two-sample and two-step Mendelian randomization (MR) analysis to evaluate the association of T2D with CAVS and the mediating effects of circulating metabolites and blood pressure using genome-wide association study (GWAS) summary statistics. The inverse variance weighted (IVW) method was used for the primary MR analysis, and comprehensive sensitivity analyses were performed to validate the robustness of the results. Our results showed that genetically predicted T2D was associated with increased CAVS risk (OR 1.153, 95% CI 1.096–1.214, *p* < 0.001), and this association persisted even after adjusting for adiposity traits in multivariable MR analysis. Furthermore, the two-step MR analysis identified 69 of 251 candidate mediators that partially mediated the effect of T2D on CAVS, including total branched-chain amino acids (proportion mediated: 23.29%), valine (17.78%), tyrosine (9.68%), systolic blood pressure (8.72%), the triglyceride group (6.07–11.99%), the fatty acid group (4.78–12.82%), and the cholesterol group (3.64–11.56%). This MR study elucidated the causal impact of T2D on CAVS risk independently of adiposity and identified potential mediators in this association pathways. Our findings shed light on the pathogenesis of CAVS and suggest additional targets for the prevention and intervention of CAVS attributed to T2D.

## 1. Introduction

Calcific aortic valve stenosis (CAVS) is the most common valvular heart disease, characterized by leaflet fibrous thickening, extracellular matrix remodeling, and calcium deposition in the valvular tissue [1]. The prevalence of CAVS is age-dependent, and thus is anticipated to increase due to global population aging [2]. The progressive impairment and deterioration of the aortic valve drive cardiac remodeling within the left ventricle, ultimately resulting in heart failure and death [3]. Currently, there are no pharmacological therapies available to delay or prevent CAVS. The only definitive treatment for patients with severe aortic stenosis is surgical aortic valve replacement (SAVR) or transcatheter aortic valve replacement (TAVI) [4,5]. Therefore, identifying independent risk factors of CAVS and patients who should be targeted with more intensive therapy has important implications.

Type 2 diabetes (T2D) has been identified as an important risk factor for cardiovascular diseases [6,7,8], which remain the leading cause of death in diabetic individuals. Several observational studies have shown a positive association between T2D and CAVS, revealing that patients with T2D have a higher incidence of CAVS and experience more serve disease progression [9,10,11,12]. Considering the high social burden of both diseases, assessing the potential effect of T2D on the development of CAVS would help to identify patients who might benefit from risk factor modification. However, the findings from observational studies cannot be directly interpreted as causation due to the uncontrolled confounding and reverse causality. Mendelian randomization (MR) is a method that utilizes genetic variants as instrumental variables (IVs) to validate the casual relationship between an exposure and an outcome [13]. Compared with observational studies, MR analysis can diminish confounding bias and circumvent reverse causality, because genetic alleles are randomly allocated and fixed at conception [14].

Here, we conducted an MR analysis to investigate the casual, independent association between T2D and CAVS, with a particular interest in identifying potential mediators and quantifying their mediation proportions in associated pathways. For mediation analysis, the two-step MR approach is not only sensitive to the causal effects of the mediators but also corrects for measurement errors [15].

## 2. Materials and Methods

### 2.1. Study Design

**Figure 1:**
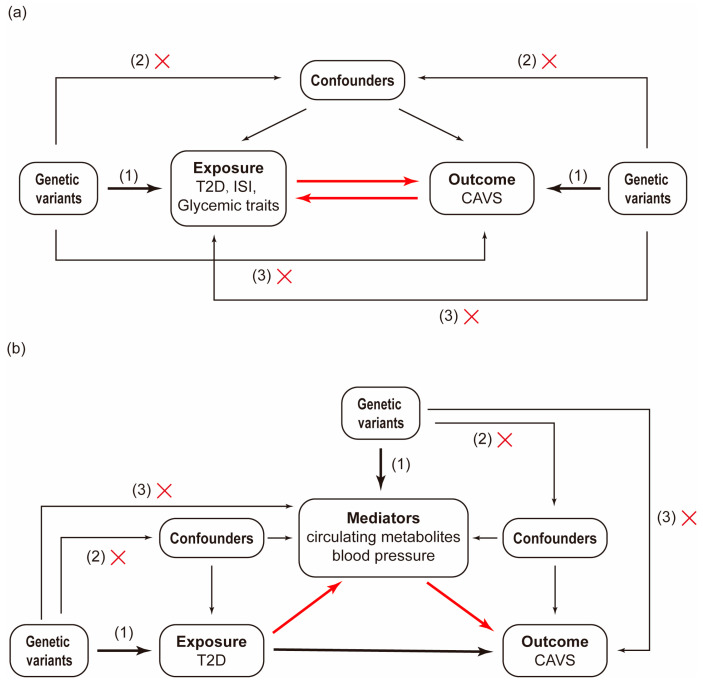
The flowchart of the study design. (**a**). Evaluate the causal relationships between T2D, ISI, glycemic traits and CAVS; (**b**). Assess the mediating roles of circulating metabolites and blood pressure in the pathway between T2D and CAVS. All MR analyses in this study were grounded on three core assumptions: (1) IVs must be significantly connected to the exposure; (2) IVs must be independent of confounders of the relationship between the exposure and outcome; and (3) IVs cannot be associated with the outcome except through the exposure. T2D, type 2 diabetes; ISI, insulin sensitivity index; CAVS, calcific aortic valve stenosis.

This MR study consisted of two stages (Figure 1). In stage 1, we performed two-sample univariable bidirectional MR analysis using genome-wide association study (GWAS) summary statistics to evaluate the causal relationships between T2D, the insulin sensitivity index (ISI), glycemic traits, and CAVS. We then conducted a multivariable MR analysis to estimate the independent effect of T2D on CAVS with adjustment for adiposity traits. In stage 2, we applied a two-step MR method to assess the mediating roles of 249 metabolic traits and two components of blood pressure in the pathway between T2D and CAVS, and calculated the mediation proportions for the identified mediators.

This MR study was performed following the latest STROBE-MR guidelines [16]. The causal effects assessed by the MR method were based on three assumptions: (1) IVs must be significantly connected to the exposure; (2) IVs must be independent of confounders of the relationship between the exposure and outcome; and (3) IVs cannot be associated with the outcome except through exposure [13]. All data and materials used in this MR study are publicly available, and specific sources can be found in the article. Informed consent and ethical approval had been obtained for the original studies [17,18,19,20,21,22,23].

### 2.2. Data Sources and Genetic Instrument Selection

CAVS-related genetic variants were extracted from the FinnGen consortium (https://r9.risteys.finngen.fi/endpoints/I9_CAVS_OPERATED, accessed on 9 March 2024), which included 9153 cases and 382,996 controls. Genetic variants associated with T2D and glycemic traits (fasting glucose, fasting insulin, and hemoglobin A1c (HbA1c)) were selected from a GWAS meta-analysis comprising 62,892 T2D cases and 596,424 controls [17], and from the Meta-Analyses of Glucose and Insulin-related traits Consortium (MAGIC) with up to 281,416 individuals without diabetes [18]. Summary-level data of ISI were acquired from the GWAS conducted by Williamson et al. in 2023 [19]. GWAS data for adiposity traits, including body mass index (BMI, *n* = 339,224) [20], waist circumference (WC, *n* = 245,746) [21], and waist-to-hip ratio (WHR, *n* = 224,459) [21], were acquired from the Genetic Investigation of Anthropometric Traits (GIANT) consortium. Summary-level genetic associations with 249 metabolic traits (165 concentrations and 84 derived ratios) were obtained from the UK Biobank study with 115,078 participants [22]. Moreover, GWAS datasets used for systolic blood pressure (SBP, *n* = 757,601) and diastolic blood pressure (DBP, *n* = 757,601) came from the International Consortium of Blood Pressure [23]. Detailed information about the data sources mentioned above is presented in Table S1.

Single nucleotide polymorphisms (SNPs) with genome-wide significance (*p* < 5 × 10^−8^) were selected from the corresponding GWASs, and were clumped using a pairwise linkage disequilibrium (LD) threshold of r2 < 0.001 in a 10,000 kb window. SNPs with palindromic alleles and those that exhibited a significant relationship (*p* < 5 × 10^−8^) with the outcome, or were not available in the outcome, were eliminated. Furthermore, F statistics were calculated utilizing the formula: F = R^2^(N − K − 1)/[K(1 − R^2^)], in which R^2^ indicates variability explained by genetic instruments, K represents the number of SNPs, and N represents the sample size. SNPs with F statistics > 10 were chosen as reliable and valid IVs to avoid weak instrument bias [24].

### 2.3. Statistical Analysis

The inverse-variance weighted (IVW) method was used to obtain the primary causal estimate, supplemented by other MR analysis methods (MR-Egger, weighted median, simple mode, and weighted mode). Odds ratios (OR) with 95% confidence intervals (CIs) were presented for the causal effects. The causal association between the exposure and outcome was deemed significant with a *p*-value < 0.05. For sensitivity analyses, we assessed the heterogeneity for the IVW estimates applying Cochran’s Q test [25] and evaluated the horizontal pleiotropy based on the intercept term in the MR-Egger regression model [26]. Additionally, we used the MR-PRESSO method to identify and adjust for any outlier SNP reflecting likely horizontal pleiotropic biases [27] and performed leave-one-out analyses to detect whether the results were influenced by any single SNP.

In two-step MR analysis, the Benjamini–Hochberg method was utilized to calculate the adjusted *p*-value to control the false discovery rate (FDR). Associations with *p*-value < 0.05 and adjusted *p*-value < 0.05 were considered statistically significant, while associations with *p*-value < 0.05 and adjusted *p*-value > 0.05 were defined as suggestive associations. The mediation proportions were computed according to the formula: (β1∗β2)/β, where β represents the total effect of T2D on CAVS, β1 represents the effect of T2D on mediators, and β2 represents the effect of mediators on CAVS. Standard errors for the mediating effects were calculated using the delta method [28].

All statistical analysis were conducted with the “TwoSampleMR” [29], “MendelianRandomization” [30], and “MR-PRESSO” [27] packages in R software, version 4.3.1.

## 3. Results

### 3.1. Causal Effect of T2D on CAVS

**Figure 2:**
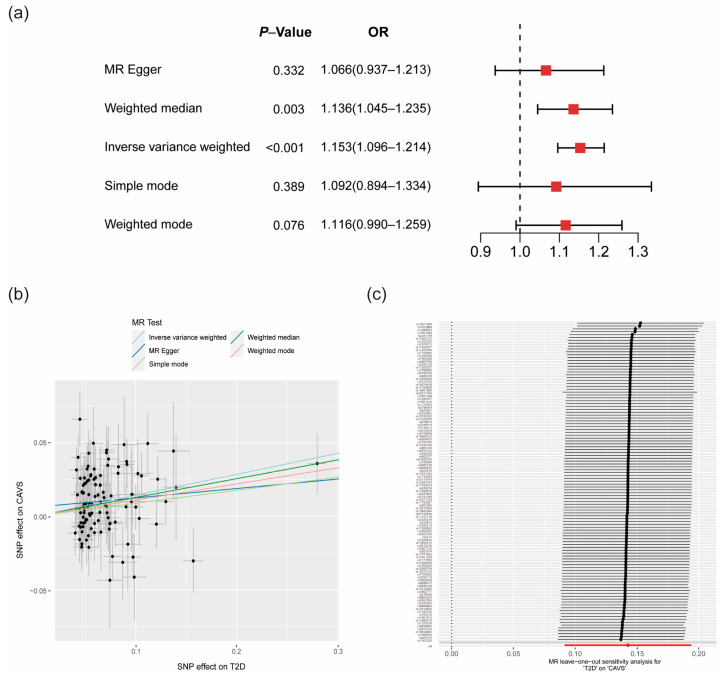
Causal association of genetically predicted T2D with CAVS risk. The forest plot (**a**) and scatter plot (**b**) to visualize the causal effect of T2D on CAVS assessed by five different MR methods; (**c**) The forest plot of leave-one-out analyses to show the influence of individual SNP on the association. OR, odds ratio; T2D, type 2 diabetes; CAVS, calcific aortic valve stenosis; SNP, single nucleotide polymorphism.

The causal association of T2D with CAVS by univariable MR analysis is shown in Figure 2a,b. The result using IVW method indicated that there was a significant causal effect of T2D on CAVS (OR 1.153, 95% CI 1.096–1.214, *p* < 0.001). A consistent estimate of effect was identified using weighted median method (OR 1.136, 95% CI 1.045–1.235, *p* = 0.003). The horizontal pleiotropy test using MR egger regression demonstrated that there was no significant bias from pleiotropy affecting the causal estimate (intercept b 0.006, *p* = 0.195). Moreover, Cochran’s Q test revealed little evidence of heterogeneity across SNPs (*p* = 0.069) (Table S2). The results of leave-one-out analyses, illustrated in Figure 2c, suggested that the association of T2D with CAVS was not driven by a single SNP.

Furthermore, we performed multivariate MR analyses to reduce the influence of potential confounders (Figure S1). As shown in the figure, the causal association between T2D and CAVS remained significant after adjustment for BMI (OR 1.105, 95% CI 1.040–1.174, *p* = 0.001), WC (OR 1.107, 95% CI 1.041–1.177, *p* = 0.001), and WHR (OR 1.088, 95% CI 1.026–1.153, *p* = 0.004).

### 3.2. Causal Effect of CAVS on T2D

To exclude the possibility of reversal causality, we conducted further analysis to examine the causal effect of CAVS on T2D (Figure S2). However, there was no evidence that genetic liability to CAVS causally increases T2D risk (IVW-OR 1.020, 95% CI 0.975–1.066, *p* = 0.389). Subsequent analyses also showed no evidence of horizontal pleiotropy (intercept b = −0.008, *p* = 0.629) or heterogeneity (*p* = 0.349) (Table S3). The leave-one-out analyses revealed that no single SNP significantly influenced the results (Figure S3).

### 3.3. Causal Effects of Glycemic Traits and Insulin Resistance on CAVS

**Figure 3:**
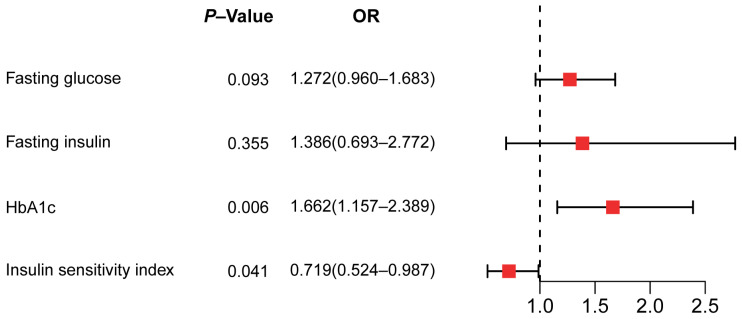
Causal associations of genetically predicted glycemic traits and insulin resistance with CAVS risk assessed by the IVW method. OR, odds ratio; HbA1c, hemoglobin A1c.

Next, we explored the association of genetically predicted glycemic traits (fasting glucose, fasting insulin, HbA1c) and insulin sensitivity index with CAVS in order to investigate underlying mechanisms related to impaired glycemic homeostasis (Figure 3).

Using the IVW method, we found that genetically predicted HbA1c was significantly related to a higher risk of CAVS (OR 1.662, 95% CI 1.157–2.389, *p* = 0.006. However, there was no convincing evidence of causal effects of fasting glucose (OR 1.272, 95% CI 0.960–1.683, *p* = 0.093) and fasting insulin (OR 1.386, 95% CI 0.693–2.772, *p* = 0.355) on CAVS. Furthermore, we also observed an association between ISI and CAVS (OR 0.719, 95% CI 0.524–0.987, *p* = 0.041), demonstrating that insulin resistance is involved in the pathological course of CAVS. Further examination revealed potential horizontal pleiotropy in SNPs related to HbA1c (intercept b 0.032, *p* = 0.007), and heterogeneity in SNPs related to fasting glucose (*p* < 0.001) or fasting insulin (*p* < 0.001) (Table S2). The results of the leave-one-out analyses are depicted in Figures S4–S7. Of note, rs1260326 showed a disproportionately high contribution to the association between fasting glucose and CAVS (Figure S4). Additionally, four potentially influential SNPs (rs459193, rs11128603, rs2972144, rs1906937) appeared to drive the causal link between ISI and CAVS (Figure S7).

### 3.4. Mediating Effects of Metabolites and Blood Pressure in the Association of T2D with CAVS

Two-step MR analyses were performed to assess the mediating roles of circulating metabolites and blood pressure using the IVW method. The causal effects of T2D on mediators, and mediators on CAVS are separately presented in Tables S4 and S5. Our results indicated that genetically instrumented T2D was causally associated with 199 candidate mediators among the 249 metabolic traits and two components of blood pressure. In subsequent MR analyses, significant associations between 126 out of the 199 candidate mediators and CAVS were observed. The mediation proportions were calculated for the 69 mediators through which indirect effects were consistent with the direct effect of T2D on CAVS. The detailed results are listed in Table 1. Notably, total branched-chain amino acids (BCAAs) mediated 23.29% of the total effect of T2D on CAVS risk, followed by valine (Val) 17.78%, ratio of omega-6 fatty acids to total fatty acids 12.82%, total lipids in very-large very-low-density lipoprotein (VLDL) 12.28%, concentration of very large VLDL particles 12.05%, and total triglycerides (TG) 11.99%. Most of these results showed no evidence of pleiotropy except for a few (Tables S6 and S7). Additionally, Cochran’s Q statistics indicated that heterogeneity from IVs might exist (Tables S8 and S9).

## 4. Discussion

This MR study, for the first time, evaluated the causal, independent effect of T2D on CAVS risk, and identified potential mediators and quantified their mediation proportions in the pathway between T2D and CAVS. The results demonstrated that genetically predicted T2D causally significantly increased CAVS risk, and the association remained consistent when adjusting for BMI, WC and WHR. Our study further revealed causal associations of genetically predicted HbA1c and ISI with CAVS, while no significant correlations were found for fasting glucose and fasting insulin. In addition, 69 potential mediators were identified to partially mediate the causal effect of T2D on CAVS, including total BCAA (proportion mediated: 23.29%), Val (17.78%), tyrosin (9.68%), SBP (8.72%), the triglyceride group (6.07–11.99%), the fatty acid group (4.78–12.82%), and the cholesterol group (3.64–11.56%). These findings provide insights into the role of T2D in the pathogenesis of CAVS and support the importance of comprehensive management strategies for patients with T2D.

T2D has long been recognized as an independent risk factor for cardiovascular diseases. Previous studies have focused on the effects of T2D on coronary heart disease, cardiomyopathy, congestive heart failure, and peripheral arterial disease [31,32]. The association between T2D and CAVS has gradually come to light recently. At the observational level, the prevalence of diabetes among patients with aortic stenosis has been shown to be higher than in the general population, with a substantially increasing trend [33,34,35]. Notably, Aronow et al. pointed out in a retrospective study on patients with mild valvular aortic stenosis that individuals with diabetes had higher annual progression in peak systolic gradient compared to those without diabetes [9]. Similar results were obtained by Kamalesh et al., who observed faster progression of aortic stenosis measured as aortic valve area in patients with diabetes [36]. In addition, Mosch et al. reported that aortic valves from diabetic patients showed more calcification compared to those from non-diabetic patients [37]. The above studies suggested that diabetes mellitus fostered the CAVS progression. Taking advantage of MR approaches to minimize the potential confounding effects, our study provided strong supporting evidence by demonstrating that T2D had a causal adverse effect on CAVS. The findings were also supported by the results of a large cohort study comprising 1.12 million individuals followed for a median of 13 years [12]. Interestingly, in another study published the same year, Testuz et al. found no impact of metabolic syndrome or diabetes on CAVS progression [38]. However, this study only analyzed fasting glucose levels, whereas it has been shown that long-term glycemic control may be of key importance [39]. A recent prospective cohort study conducted by Hwang et al. in patients with mild to moderate aortic stenosis revealed that the mean HbA1c level during followed-up was significantly related to the disease progression rate [40]. Consistently, our analysis also indicated that genetic liability to HbA1c, rather than fasting glucose or fasting insulin, was linked to a higher risk of CAVS, further supporting the causal relationship between T2D and CAVS. Additionally, insulin resistance was shown to play an important role in the development of aortic valve calcification and the progression of aortic stenosis [41,42,43], and the association between insulin resistance and CAVS was confirmed in our study as well.

Given that T2D is a complex metabolic disorder, we specifically explored whether circulating metabolites serve as causal mediators that modulate the relationship between T2D and CAVS. Interestingly, among the 249 metabolic traits which represent the metabolism of glucose, amino acids, and lipids, we identified a total of 68 mediators, with the majority being associated with lipid metabolism. Previous studies have observed lipid and amino acid metabolism disturbances mainly in individuals with T2D, likely as a consequence of increased insulin resistance [44,45,46]. During insulin-resistance states such as T2D, the control of hepatic glucose production by insulin is defective yet the lipid promoting effects of insulin are maintained, leading to hyperglycemia and hypertriglyceridemia [47]. In addition, augmentation in the serum concentration of BCAAs (including leucine, valine, isoleucine) is closely related to insulin resistance, diabetes mellitus, and its complications [44,48,49]. Our study, along with a recent MR analysis [50], has affirmed the metabolic features of genetic liability to T2D, despite the presence of pleiotropy in some results. With regard to the roles of potential mediators in CAVS, we unsurprisingly revealed that VLDL, LDL, and their fine subclasses and compositions were causally associated with CAVS, which is in line with the results from previous studies [51,52]. A recent study conducted by Lee et al. pointed the association of LDL with increased valvular lipid and macrophage accumulation through single-cell transcriptomics [53]. Moreover, we also found the protective effect of high density lipoprotein (HDL) on CAVS and this could be interpreted by the reverse cholesterol transport (RCT) hypothesis [54]. This hypothesis prompted that HDL acts as the receptor and carrier of cholesterol, facilitating its efflux from peripheral tissue and delivering it to the liver for further metabolism and excretion. Our findings also highlighted total TG and TGs in various lipoproteins, even in HDL, as risk factors for CAVS among patients with T2D, emphasizing the importance of controlling triglyceride levels in preventing CAVS. It is also reasonable to assume that TG is responsible for HDL dysfunction [55]. Further investigation of lipoprotein compositions and mechanisms could be useful in understanding pathways between diabetes and aortic valve stenosis. Additionally, polyunsaturated fatty acids are reported to have cardiovascular protections and have been confirmed by observational studies [56,57,58]. Indeed, our study suggested that supplement with omega-6 fatty acids to increase the ration of omega-6 fatty acids to total fatty acids and the ratio of omega-6 to omega-3 fatty acids in diabetic patients may have a potential benefit in reducing CAVS risk. However, it is also fascinating to observe that a higher genetically predicted omega-3 fatty acid concentration was related to a higher risk of CAVS, which contradicts the result of a large randomized controlled trial [59]. Previous MR studies examined the associations between omega-3 fatty acid compositions and CAVS in depth, and showed the detrimental effects of docosahexaenoic acid (DHA), decosapentaenoic acid (DPA), and eicosapentaenoic acid (EPA), alongside the protective effect of α-linolenic acid (ALA) [60,61]. Nonetheless, studies focusing on amino acids (such as total BCAAs, Tyr, or Val) and CAVS are limited. As the total BCAAs accounted for the highest mediating effect, it is essential to explore its role in the pathogenesis of CAVS in future studies. Taken together, our findings provide new evidence of metabolic pathways linking T2D to CAVS, which require further research.

In addition, we investigated the mediating role of blood pressure in the association of T2D with CAVS, considering that hypertension is particularly prevalent in patients with T2D [62]. We unexpectedly found that SBP mediated 8.72% of the total effect of T2D on CAVS risk. Recently, studies have found causal relationships between blood pressure and metabolic risk factors such as obesity, dyslipidemia, diabetes mellitus, and insulin resistance [63,64,65], and elevated blood pressure has shown to increase susceptibility to valvular inflammation, extracellular matrix remodeling, and aortic stenosis [66,67,68], which is in agreement with our results. Furthermore, in one retrospective study, angiotension converting enzyme inhibitors have been documented to reduce the accumulation of aortic valve calcium in patients with aortic stenosis [69]. This further supports the possibility that the association between T2D and CAVS may be partly because of the increase in SBP and implicates that management of blood pressure is an important target in the prevention of CAVS.

Our study comprehensively estimated the genetic relationship between T2D, glycemic traits and CAVS, and the results revealed that genetic liability to T2D, increased HbA1c and decreased ISI are risk factors for CAVS. In addition, we found several circulating metabolites and SBP to mediate the causal pathway between T2D and CAVS, suggesting that early interventions with these factors may reduce the incidence of CAVS. Further research is needed to explore the in-depth mechanisms.

This MR study provided reliable and novel evidence for a causal effect of T2D on CAVS risk and yielded insights into potential pathways mediated by circulating metabolites and blood pressure. The utilization of MR method and summary statistics from large-scale GWASs helped mitigate potential confounding bias and ensure credible causal inference. In addition, several methods were employed to validate the robustness and reliability of the MR results. However, there are still some limitations that need to be emphasized. First, to ensure consistency in genetic background, this MR study was primarily restricted to individuals of European ancestry, limiting the generalizability of our findings to other populations. Second, although we strived to minimize pleiotropy, it is impossible to completely eliminate all instances of pleiotropy in MR analysis. Unrecognized pathways and confounding factors between the exposure and outcome may still exist, potentially introducing biases into our results. Third, this analysis was conducted with summary-level data, restricting our capacity to perform subgroup analysis, for example, by gender or age.

## 5. Conclusions

In conclusion, this study elucidated a causal adverse impact of T2D on CAVS risk independent of adiposity, and both higher HbA1c and lower ISI were associated with CAVS. In addition, the causal relationship between T2D and CAVS was found to be mediated in part by circulating metabolites and blood pressure. Our findings shed light on the pathogenesis of CAVS and imply additional targets for the prediction, prevention and intervention of CAVS among patients with T2D.

## Figures and Tables

**Table 1:** The mediating effects and mediation proportions of the mediators in the causal relationship between T2D and CAVS. A total of 69 out of the 251 candidate mediators were selected by two-step MR analysis, and their mediating effects and mediation proportions were calculated.

Category	Mediator	β1 (95% CI)	β2 (95% CI)	β1∗β2 (95% CI)	β1∗β2/β (%)
Amino acids	Total_BCAA	0.075 (0.056 to 0.093)	0.445 (0.054 to 0.837)	0.033 (0.003 to 0.064)	23.288
	Tyr	0.051 (0.031 to 0.071)	0.271 (0.081 to 0.461)	0.014 (0.002 to 0.025)	9.684
	Val	0.075 (0.056 to 0.093)	0.340 (0.022 to 0.658)	0.025 (0.001 to 0.050)	17.782
Blood pressure	SBP	0.541 (0.366 to 0.716)	0.023 (0.016 to 0.030)	0.012 (0.007 to 0.018)	8.716
Cholesterol	L_HDL_CE_pct	−0.068 (−0.089 to −0.047)	−0.155 (−0.299 to −0.012)	0.011 (0.000 to 0.021)	7.408
	L_VLDL_C	0.040 (0.019 to 0.061)	0.255 (0.109 to 0.402)	0.010 (0.002 to 0.018)	7.158
	L_VLDL_CE	0.027 (0.006 to 0.048)	0.324 (0.180 to 0.469)	0.009 (0.001 to 0.017)	6.094
	L_VLDL_FC	0.052 (0.030 to 0.074)	0.274 (0.132 to 0.416)	0.014 (0.005 to 0.024)	9.965
	M_HDL_C_pct	−0.057 (−0.077 to −0.036)	−0.191 (−0.319 to −0.062)	0.011 (0.002 to 0.019)	7.556
	M_HDL_CE_pct	−0.055 (−0.077 to −0.034)	−0.214 (−0.335 to −0.092)	0.012 (0.004 to 0.020)	8.278
	M_LDL_FC_pct	−0.075 (−0.095 to −0.055)	−0.158 (−0.274 to −0.042)	0.012 (0.003 to 0.021)	8.3
	S_HDL_C_pct	−0.052 (−0.072 to −0.031)	−0.156 (−0.294 to −0.019)	0.008 (0.000 to 0.016)	5.656
	S_HDL_CE_pct	−0.044 (−0.064 to −0.024)	−0.208 (−0.340 to −0.076)	0.009 (0.002 to 0.016)	6.419
	S_LDL_CE_pct	0.025 (0.007 to 0.044)	0.205 (0.077 to 0.334)	0.005 (0.000 to 0.010)	3.642
	S_LDL_FC_pct	−0.069 (−0.089 to −0.049)	−0.171 (−0.284 to −0.059)	0.012 (0.003 to 0.020)	8.279
	VLDL_FC	0.028 (0.006 to 0.049)	0.309 (0.176 to 0.442)	0.009 (0.001 to 0.016)	6.033
	XL_VLDL_C	0.044 (0.024 to 0.064)	0.317 (0.169 to 0.465)	0.014 (0.005 to 0.023)	9.804
	XL_VLDL_CE	0.027 (0.006 to 0.048)	0.264 (0.120 to 0.408)	0.007 (0.000 to 0.014)	5.004
	XL_VLDL_FC	0.056 (0.035 to 0.077)	0.296 (0.152 to 0.440)	0.016 (0.006 to 0.027)	11.564
	XXL_VLDL_C	0.061 (0.040 to 0.081)	0.259 (0.115 to 0.402)	0.016 (0.005 to 0.026)	11.001
	XXL_VLDL_CE	0.058 (0.037 to 0.079)	0.254 (0.126 to 0.383)	0.015 (0.005 to 0.024)	10.327
	XXL_VLDL_FC	0.065 (0.044 to 0.085)	0.202 (0.059 to 0.345)	0.013 (0.003 to 0.023)	9.16
Fatty acids	MUFA	0.047 (0.026 to 0.067)	0.305 (0.164 to 0.446)	0.014 (0.005 to 0.023)	9.972
	MUFA_pct	0.062 (0.041 to 0.083)	0.201 (0.049 to 0.353)	0.013 (0.002 to 0.023)	8.764
	Omega_3	0.022 (0.004 to 0.040)	0.312 (0.218 to 0.407)	0.007 (0.001 to 0.013)	4.783
	Omega_6_by_Omega_3	−0.031 (−0.048 to −0.013)	−0.373 (−0.524 to −0.222)	0.011 (0.003 to 0.020)	8.052
	Omega_6_pct	−0.062 (−0.083 to −0.040)	−0.297 (−0.473 to −0.120)	0.018 (0.005 to 0.031)	12.821
	SFA	0.028 (0.008 to 0.047)	0.331 (0.164 to 0.498)	0.009 (0.001 to 0.017)	6.39
	SFA_pct	0.031 (0.013 to 0.050)	0.454 (0.131 to 0.777)	0.014 (0.001 to 0.028)	9.901
	Total_FA	0.026 (0.008 to 0.044)	0.290 (0.149 to 0.430)	0.008 (0.001 to 0.014)	5.331
Lipoprptein particle concentration	L_VLDL_P	0.058 (0.037 to 0.079)	0.287 (0.140 to 0.435)	0.017 (0.006 to 0.027)	11.672
	S_VLDL_P	0.028 (0.008 to 0.048)	0.276 (0.149 to 0.403)	0.008 (0.001 to 0.014)	5.389
	VLDL_P	0.023 (0.003 to 0.044)	0.326 (0.197 to 0.455)	0.008 (0.000 to 0.015)	5.355
	XL_VLDL_P	0.062 (0.041 to 0.084)	0.276 (0.150 to 0.403)	0.017 (0.007 to 0.027)	12.049
	XXL_VLDL_P	0.067 (0.046 to 0.088)	0.246 (0.103 to 0.389)	0.017 (0.006 to 0.028)	11.605
Phospholipids	L_VLDL_PL	0.057 (0.036 to 0.077)	0.269 (0.134 to 0.404)	0.015 (0.006 to 0.025)	10.668
	L_VLDL_PL_pct	0.036 (0.015 to 0.057)	0.227 (0.097 to 0.356)	0.008 (0.001 to 0.015)	5.748
	S_LDL_PL_pct	−0.033 (−0.052 to −0.014)	−0.212 (−0.346 to −0.077)	0.007 (0.001 to 0.013)	4.917
	VLDL_PL	0.028 (0.007 to 0.049)	0.287 (0.153 to 0.421)	0.008 (0.001 to 0.015)	5.629
	XL_VLDL_PL	0.058 (0.037 to 0.079)	0.281 (0.139 to 0.423)	0.016 (0.006 to 0.027)	11.401
	XXL_VLDL_PL	0.068 (0.047 to 0.089)	0.221 (0.073 to 0.369)	0.015 (0.004 to 0.026)	10.547
	XXL_VLDL_PL_pct	0.035 (0.017 to 0.053)	0.217 (0.062 to 0.372)	0.008 (0.001 to 0.014)	5.357
Total lipids	L_VLDL_L	0.058 (0.037 to 0.079)	0.292 (0.138 to 0.447)	0.017 (0.006 to 0.028)	11.828
	S_VLDL_L	0.025 (0.005 to 0.044)	0.280 (0.154 to 0.407)	0.007 (0.001 to 0.013)	4.88
	VLDL_L	0.044 (0.024 to 0.064)	0.314 (0.175 to 0.452)	0.014 (0.005 to 0.023)	9.725
	XL_VLDL_L	0.062 (0.041 to 0.084)	0.281 (0.151 to 0.411)	0.018 (0.007 to 0.028)	12.282
	XXL_VLDL_L	0.066 (0.045 to 0.087)	0.192 (0.033 to 0.351)	0.013 (0.001 to 0.024)	8.885
Triglycerides	HDL_TG	0.048 (0.030 to 0.066)	0.227 (0.131 to 0.324)	0.011 (0.005 to 0.017)	7.641
	IDL_TG	0.035 (0.016 to 0.054)	0.249 (0.118 to 0.381)	0.009 (0.002 to 0.015)	6.066
	L_HDL_TG_pct	0.059 (0.040 to 0.079)	0.184 (0.075 to 0.293)	0.011 (0.003 to 0.018)	7.646
	L_LDL_TG	0.036 (0.017 to 0.055)	0.258 (0.129 to 0.387)	0.009 (0.003 to 0.016)	6.56
	L_VLDL_TG	0.064 (0.042 to 0.085)	0.257 (0.105 to 0.409)	0.016 (0.005 to 0.028)	11.521
	LDL_TG	0.039 (0.020 to 0.058)	0.253 (0.131 to 0.375)	0.010 (0.003 to 0.017)	6.938
	M_HDL_TG	0.052 (0.033 to 0.070)	0.214 (0.116 to 0.313)	0.011 (0.005 to 0.018)	7.761
	M_HDL_TG_pct	0.051 (0.031 to 0.071)	0.187 (0.067 to 0.308)	0.010 (0.002 to 0.017)	6.692
	M_LDL_TG	0.044 (0.024 to 0.064)	0.247 (0.117 to 0.377)	0.011 (0.003 to 0.019)	7.594
	M_VLDL_TG	0.052 (0.030 to 0.073)	0.303 (0.167 to 0.440)	0.016 (0.006 to 0.025)	10.969
	S_HDL_TG	0.063 (0.043 to 0.083)	0.218 (0.107 to 0.329)	0.014 (0.005 to 0.022)	9.624
	S_HDL_TG_pct	0.057 (0.036 to 0.078)	0.173 (0.061 to 0.284)	0.010 (0.002 to 0.017)	6.883
	S_LDL_TG	0.055 (0.035 to 0.076)	0.276 (0.145 to 0.406)	0.015 (0.006 to 0.024)	10.664
	S_LDL_TG_pct	0.074 (0.052 to 0.095)	0.165 (0.031 to 0.299)	0.012 (0.002 to 0.023)	8.51
	S_VLDL_TG	0.059 (0.037 to 0.080)	0.235 (0.124 to 0.346)	0.014 (0.005 to 0.022)	9.637
	TG_by_PG	0.067 (0.046 to 0.088)	0.161 (0.037 to 0.285)	0.011 (0.002 to 0.020)	7.526
	Total_TG	0.061 (0.040 to 0.081)	0.282 (0.155 to 0.408)	0.017 (0.007 to 0.027)	11.989
	VLDL_TG	0.063 (0.042 to 0.084)	0.260 (0.129 to 0.392)	0.016 (0.006 to 0.026)	11.485
	XL_HDL_TG_pct	0.065 (0.045 to 0.085)	0.198 (0.081 to 0.316)	0.013 (0.004 to 0.022)	9.035
	XL_VLDL_TG	0.070 (0.048 to 0.092)	0.232 (0.092 to 0.372)	0.016 (0.005 to 0.027)	11.372
	XS_VLDL_TG	0.045 (0.024 to 0.065)	0.217 (0.092 to 0.342)	0.010 (0.002 to 0.017)	6.813
	XXL_VLDL_TG	0.067 (0.046 to 0.089)	0.186 (0.014 to 0.359)	0.013 (0.000 to 0.025)	8.785

Abbreviations: BCAA, branched-chain amino acid; Tyr, tyrosine; Val, valine; SBP, systolic blood pressure; HDL, high density lipoprotein; IDL, intermediate density lipoprotein; LDL, low density lipoprotein; VLDL, very low density lipoprotein; TG, triglyceride; PG, phosphoglyceride; FC, free cholesterol; CE, cholesteryl ester; C, cholesterol; PL, phospholipid; L, lipid; MUFA, monounsaturated fatty acid; SFA, saturated fatty acid; FA, fatty acid; XXL, extremely large; XL, very large; L, large; M, medium; S, small; XS, very small; pct, percent; Omega_6_by_Omega_3, ratio of omega-6 fatty acids to omega-3 fatty acids; TG by PG, ratio of triglycerides to phosphoglycerides; β, the effect of type 2 diabetes on calcific aortic valve stenosis; β1, the effect of type 2 diabetes on mediators; β2, the effect of mediators on calcific aortic valve stenosis; CI, confidence interval. The meanings of specific abbreviations for mediators can be found in Table S10.

## Data Availability

Publicly available datasets were analyzed in this study. The original contributions presented in the study are included in the article. Further inquiries can be directed to the corresponding author.

## Supplementary Materials

The following supporting information can be downloaded at: https://www.mdpi.com/article/10.3390/metabo14070385/s1, Figure S1: multivariable MR analyses of the association of genetically predicted T2D with the risk of CAVS after adjusting for BMI, WC and WHR; Figure S2: association of genetically predicted CAVS with the risk of T2D assessed by five different MR methods; Figure S3: forest plot of leave-one-out analyses to show the influence of individual SNP on the association of CAVS on T2D; Figure S4: forest plot of leave-one-out analyses to show the influence of individual SNP on the association of fasting glucose on T2D; Figure S5: forest plot of leave-one-out analyses to show the influence of individual SNP on the association of fasting insulin on T2D; Figure S6: forest plot of leave-one-out analyses to show the influence of individual SNP on the association of HbA1c on T2D; Figure S7: forest plot of leave-one-out analyses to show the influence of individual SNP on the association of insulin sensitivity index on T2D; Table S1: GWAS data sources of the MR study; Table S2: heterogeneity and horizontal pleiotropy tests for the effects of T2D, glycemic traits and IR on CAVS; Table S3: heterogeneity and horizontal pleiotropy tests for the effect of CAVS on T2D; Table S4: the effects of T2D on circulating metabolites and blood pressure; Table S5: the effects of circulating metabolites and blood pressure on CAVS; Table S6: pleiotropy tests for the effects of T2D on mediators; Table S7: pleiotropy tests for the effects of mediators on CAVS; Table S8: heterogeneity tests for the effects of T2D on mediators; Table S9: heterogeneity tests for the effects of mediators on CAVS; Table S10: the meanings of specific abbreviations for mediators.
