# Supplementary figures and images for: Type 2 Diabetes, Circulating Metabolites, and Calcific Aortic Valve Stenosis: A Mendelian Randomization Study
Source: Metabolites. 2024 Jul 13;14(7):385. doi: 10.3390/metabo14070385 (PMC11278608; doi:10.3390/metabo14070385)

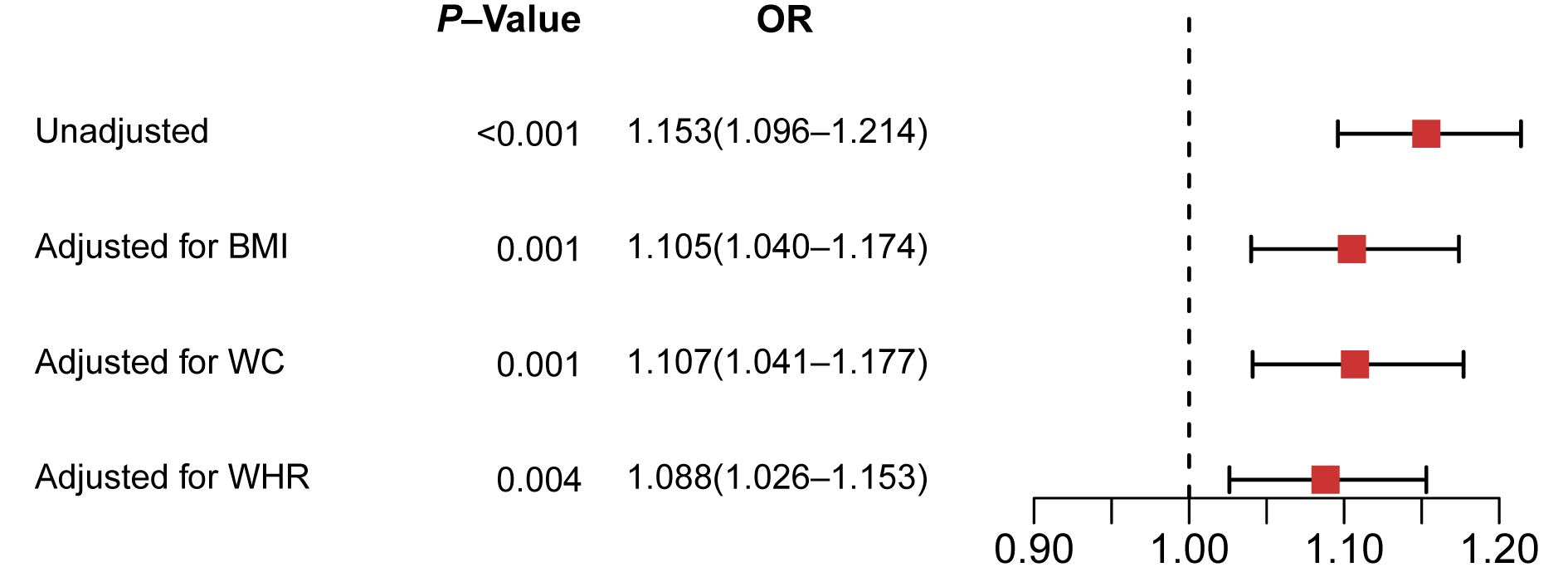

Supplement: Supplementary file 1 [file metabolites-14-00385-s001.zip › Figure S1.tif]

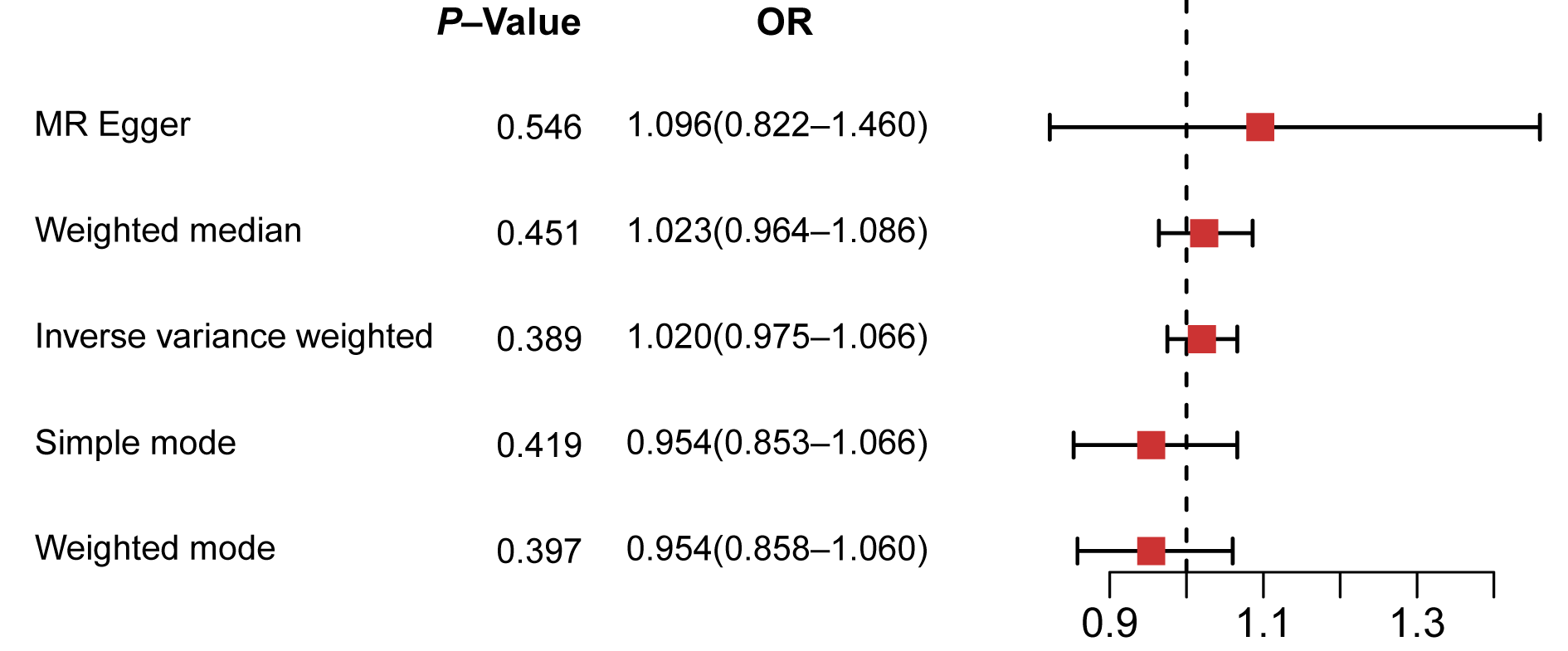

Supplement: Supplementary file 1 [file metabolites-14-00385-s001.zip › Figure S2.tif]

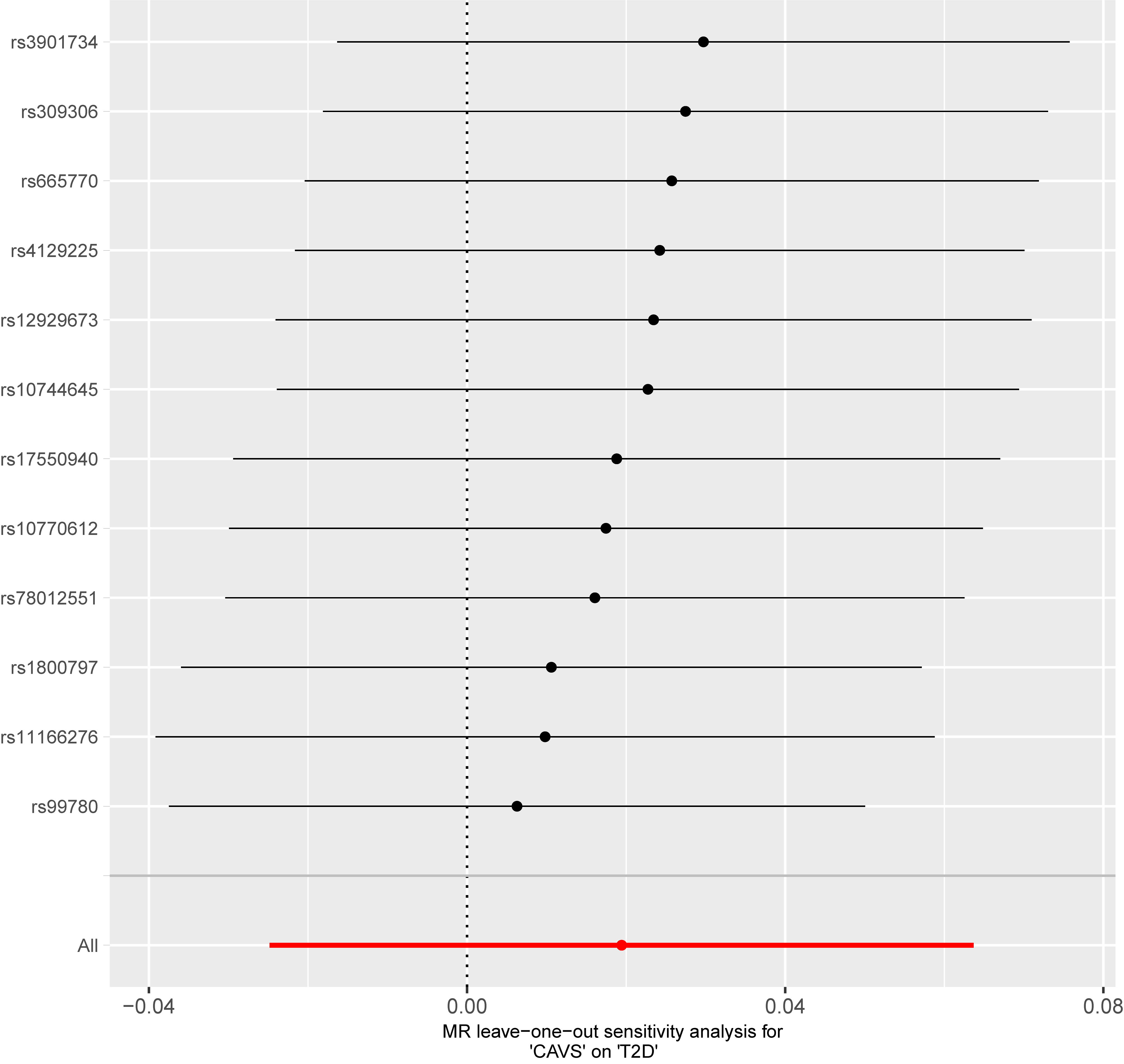

Supplement: Supplementary file 1 [file metabolites-14-00385-s001.zip › Figure S3.tif]

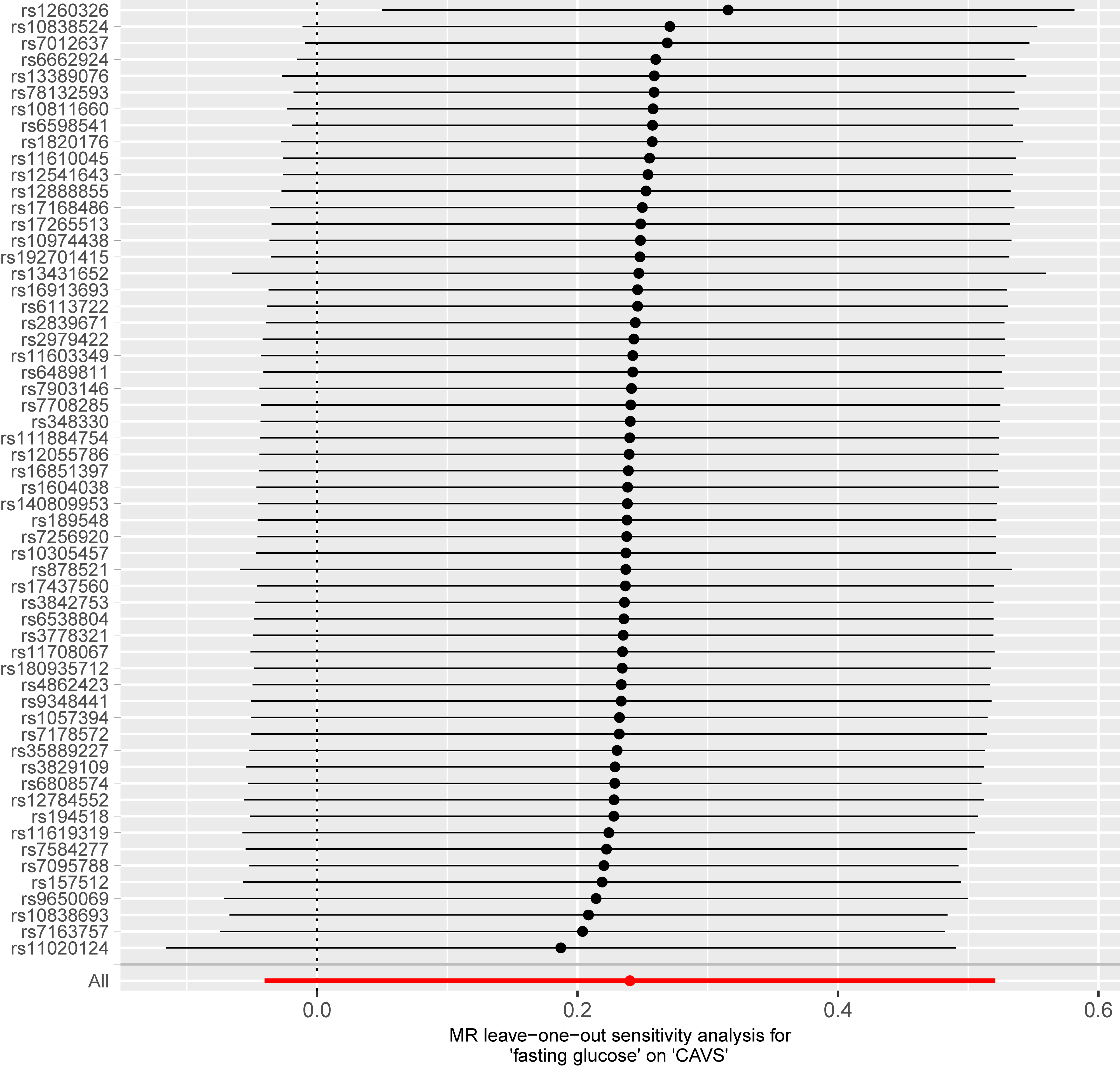

Supplement: Supplementary file 1 [file metabolites-14-00385-s001.zip › Figure S4.tif]

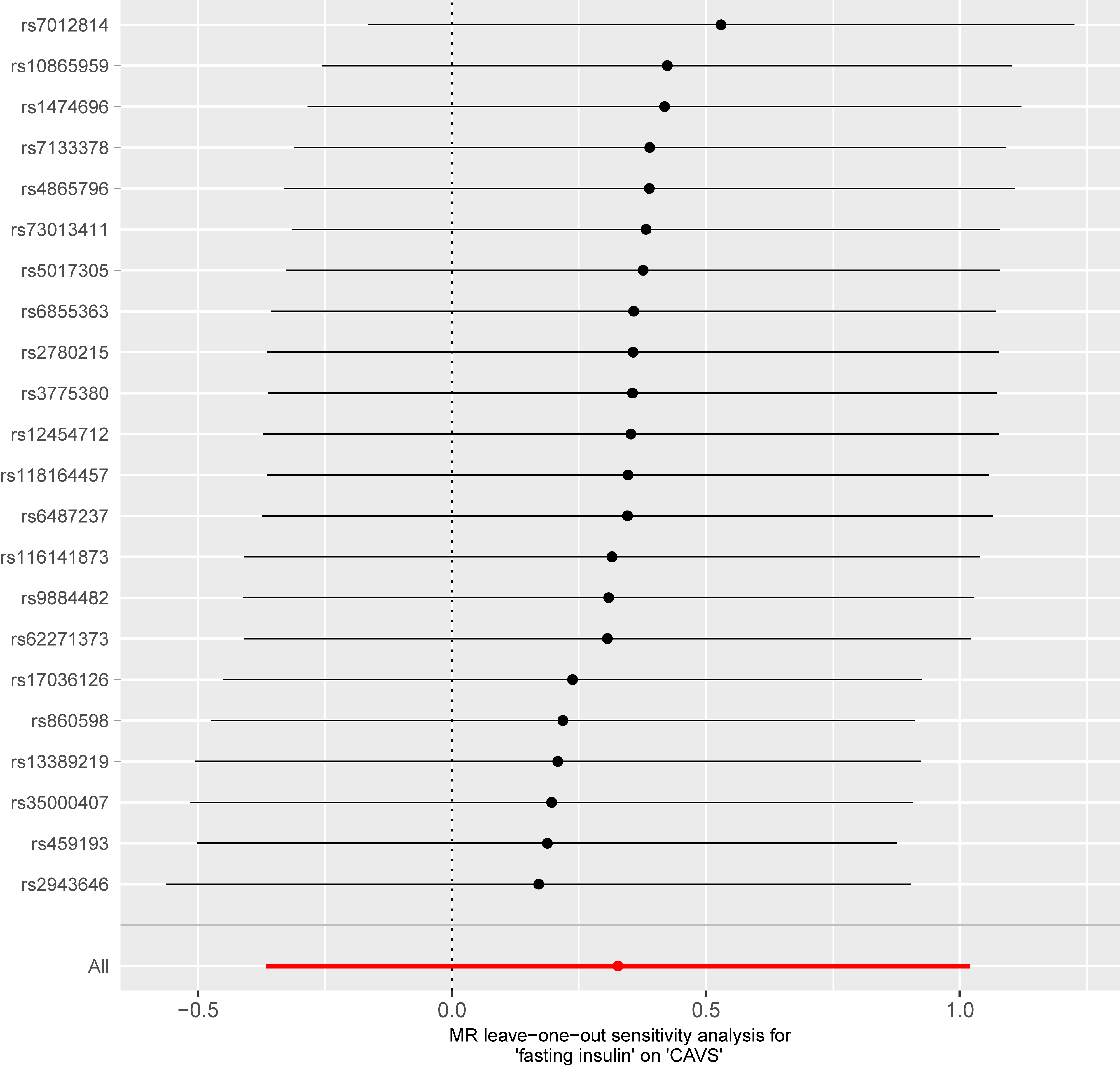

Supplement: Supplementary file 1 [file metabolites-14-00385-s001.zip › Figure S5.tif]

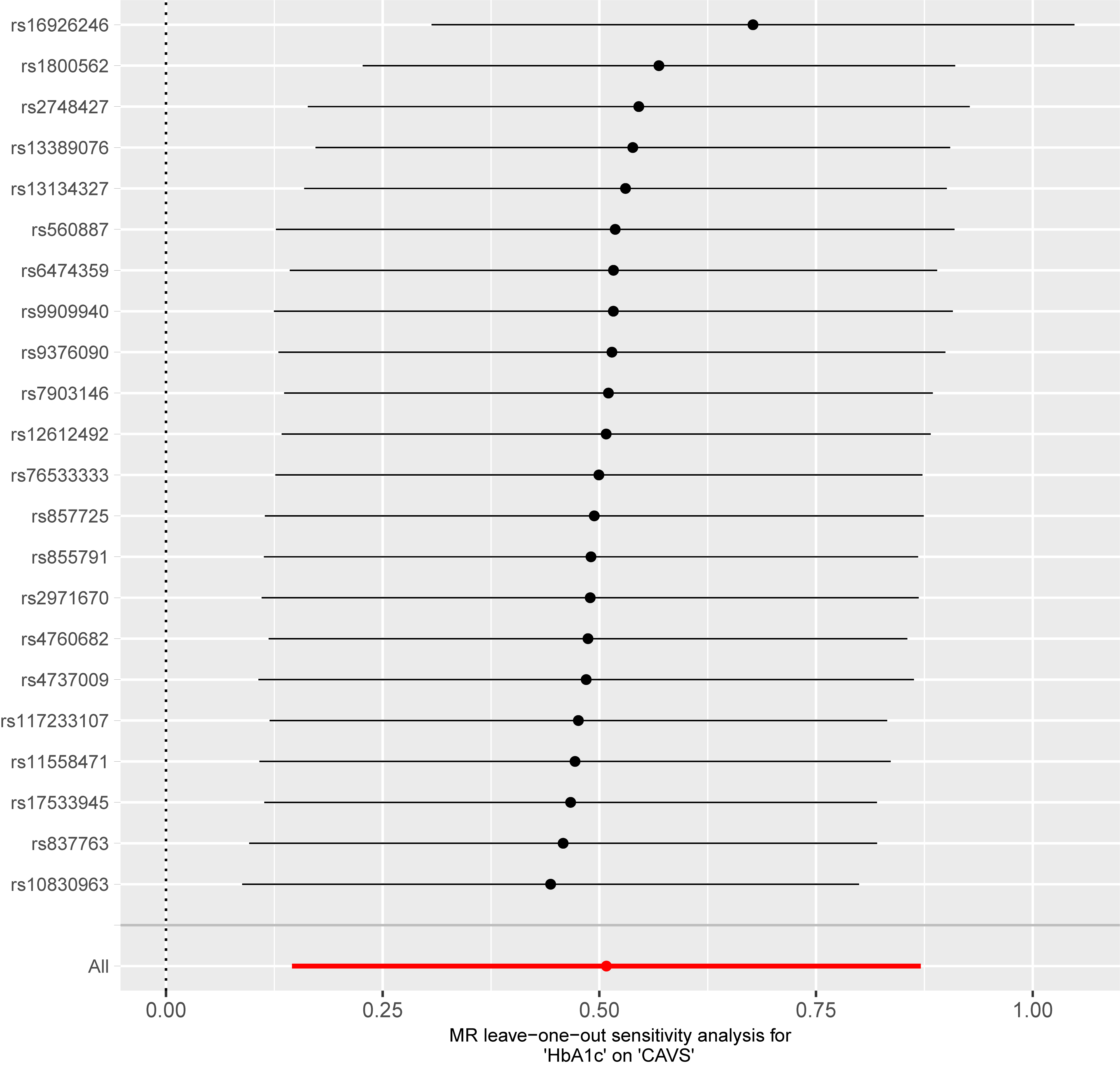

Supplement: Supplementary file 1 [file metabolites-14-00385-s001.zip › Figure S6.tif]

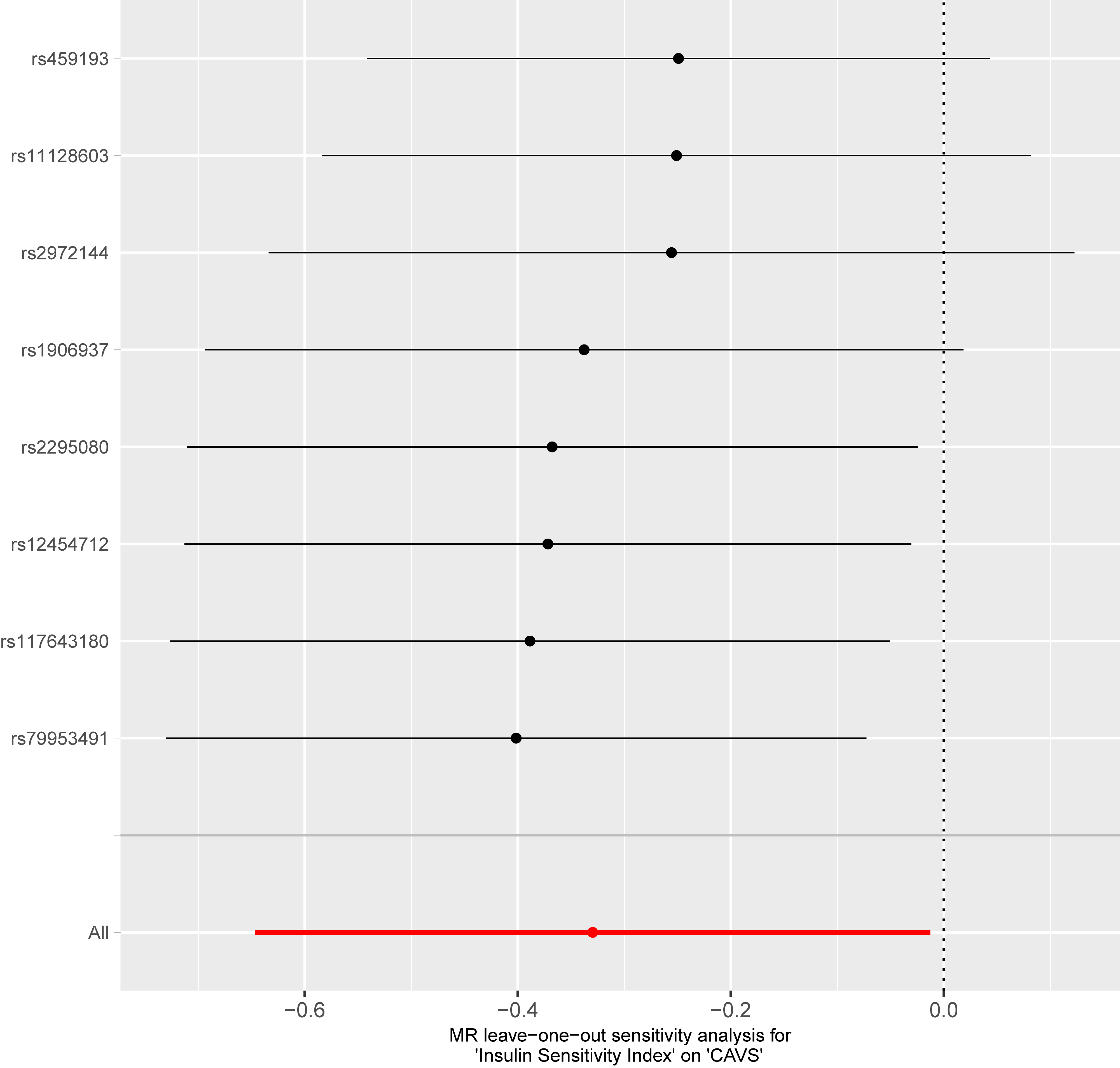

Supplement: Supplementary file 1 [file metabolites-14-00385-s001.zip › Figure S7.tif]
